# Interviewing children: the impact of the COVID-19 quarantine on children’s perceived psychological distress and changes in routine

**DOI:** 10.1186/s12887-021-02704-1

**Published:** 2021-05-13

**Authors:** G. Segre, R. Campi, F. Scarpellini, A. Clavenna, M. Zanetti, M. Cartabia, M. Bonati

**Affiliations:** Laboratory for Mother and Child Health, Department of Public Health, Istituto di Ricerche Farmacologiche Mario Negri IRCCS, Via Mario Negri 2, 20156 Milan, Italy

**Keywords:** COVID-19 quarantine, Children, Psychological distress

## Abstract

**Background:**

The COVID-19 outbreak has resulted in governments implementing disease containment measures such as school closures, social distancing, and home quarantine.

To date, only a few studies have drawn attention to the psychological impact of lockdown on Italian children’s mental health. The present study aimed to investigate the psychological distress (anxiety and mood symptoms) and perceived changes in routine among Italian primary and middle school students during the COVID-19 quarantine.

**Methods:**

This interview study was performed between the 18th of May and 7th of June 2020: it involved a sample of 82 children and adolescents living in Milan (Italy), attending primary and middle school (aged 6 to 14 years), and their parents.

**Results:**

Almost 30 % of the subjects reported having struggled to adjust to home learning. 36 responders completely changed their dietary habits during the lockdown: they were not eating the same amount of food and were consuming more junk food. Sleep habits were also affected by the lockdown measures: 28 % of the sample had difficulties sleeping and wished to sleep in their parents’ bed. Concerning psychological distress, 64 (78 %) children and adolescents had anxiety symptoms; 43.9 % of the students reported significant mood symptoms.

**Conclusions:**

Children are not indifferent to the dramatic impact of the COVID-19 epidemic: our data confirm their difficulties in adapting to the quarantine measures. The effects of stress exposure may not manifest later on during the children’s development, and, for this reason, it would be interesting to follow up on these participants to improve our understanding of how long these outcomes may last.

**Supplementary Information:**

The online version contains supplementary material available at 10.1186/s12887-021-02704-1.

## Background

In December 2019, the outbreak of a new strain of coronavirus disease, caused by severe acute respiratory syndrome coronavirus 2 (SARS-CoV-2 virus) was first reported in Wuhan, (China) and spread across the world within a short time; the World Health Organization declared it as a pandemic (COVID-19) on March 12, 2020 [1].

This pandemic has resulted in governments implementing disease containment measures such as school closures, social distancing, and home quarantine.

In Lombardy, the most affected Italian region, the first school closure began the 21st of February 2020, and, on 5 March 2020, all schools in Italy were closed and students isolated at home for the rest of the academic year, with schooling shifted to home based distance-learning models. The closure of schools was the first mass intervention taken towards a target population. School activity was the first to be suspended and the last to be resumed, by not considering how to mitigate the negative impacts of lockdown. There is still significant controversy about the role of children in spreading the virus, also at school: evidence is emerging that children may be significantly less likely to become infected than adults, and do not appear to be super spreaders [2]. On the one side, it is suggested that children may play and attenuating role both with respect to epidemiological and clinical dynamics. On the other side, some of these effects may be age dependent, with younger children more likely candidates for a lesser role in transmission [3]. According to the United Nations Educational, Scientific and Cultural Organization [4] due to the COVID-19 schools have been suspended nationwide in 188 countries: affecting more than more than 1.5 billion children and adolescents worldwide, who found themselves isolated at home. Moreover, the lockdown and school closure may have negative consequences on children, affecting their social life, their education, and their mental health [5]. The COVID-19 pandemic may worsen existing mental health problems and lead to more cases among children and adolescents because of the unique combination of uncertainty, anxiety, fear of becoming ill or seeing a loved one become sick, loss of our everyday routines, difficulties in maintaining social connection, and economic recession [6].

After the H1N1 and SARS epidemics, post-traumatic stress is estimated to be four times higher in children who have been in quarantine compared to those who have not, and their likelihood of presenting acute stress disorder, adjustment disorder, and grief is also higher [7].

A recent review highlighted that children and adolescents are probably more likely to experience high rates of depression and anxiety during and after enforced isolation ends [8]. The authors found a clear association between loneliness and mental health problems, mostly depression, in children and adolescents. Loneliness was associated with future mental health problems up to 9 years later.

A Chinese survey [9] conducted during COVID-19 directly involved primary school students and reported higher rates of depressive (22.6 %) and anxiety (18.9 %) symptoms compared with the prevalence in other surveys.

Italy was the first European country to implement a national lockdown to contain the spread of severe coronavirus disease 19 (COVID-19) and related strict domestic quarantine policies.

To date, only a few studies [10–13] have drawn attention to the psychological impact of lockdown on Italian children’s mental health. A recent study has examined the psychological effects of the quarantine in youth from Italy and Spain. Data were collected through a survey completed by parents and found that their children had different symptoms such as: difficulty concentrating (76.6 %), boredom (52 %), irritability (39 %), restlessness (38.8 %), nervousness (38 %), feelings of loneliness (31.3 %), uneasiness (30.4 %), and worries (30.1 %). Moreover, the results show that children of both countries used monitors more frequently, spent less time doing physical activity, and slept more hours during the quarantine (10). Similarly, another Italian study [13] suggested that during the lockdown children exhibited a marked delay in sleep timing and a mild worsening in sleep quality. They were less prone to respect daily routines or to keep track of the passage of time. An increase in emotional, conduct and hyperactive symptoms in children, together with regressive behaviours was reported, which was predicted by the change in sleep quality, boredom, and mothers’ psychological difficulties.

The social distancing and stay-at-home orders issued in cities across the globe obviously reduce the opportunities for physical activity among children, particularly for children in urban areas living in small apartments. Isolation and shielding could result in increased sedentary behaviours and food consumption, which are likely to impact weight and consequently health and sleep over time [14, 15].

During the pandemic, the prevalence of physically inactive students increased extensively, from 21.3 to 65.6 %. Overall screen time increased considerably during the pandemic and screen time during leisure was also prolonged, indicating that nearly a quarter of students engaged in long screen time for leisure [9].

To date, the majority of knowledge available from research related to the health needs and experiences of young children has been based on the perspectives of parents and/or paediatric health professionals. A growing number of researchers in the health care field have begun to capture the children’s perspectives through interviews [16, 17].

For this reason, we decided to directly involve children and adolescents with video-interviews to convey their experiences and to more fully understand their needs.

To our knowledge, this is the first study that directly investigates the effects of the COVID-19 lockdown on children’s and adolescents’ perceived changes in routine and mental health from their own perspective. In particular, the added value of the study is the fact that it directly involved students with video-interviews, letting them share their needs and opinions.

The present study aims to analyse the impact of the quarantine on students’ life in Milan, one of the Italian cities most affected by COVID-19. In particular, we focused our attention on their perception of changes in routine during the lockdown: distance learning, eating and sleeping habits. Psychological distress such as anxiety and mood symptoms, disease concern, regressive behaviours, and fears were examined. We hypothesized that quarantine period and school closure could have a negative impact on perceived changes in routine and on children’s emotional and behavioral well-being. Moreover we aimed to explore what are the things that children missed the most and what they expected to do as soon as quarantine gets over, in order to highlight their priorities.

## Methods

### Study design

This interview study was performed between the 18th of May and 7th of June 2020, for a total of 3 weeks. 18th of May was a meaningful date in Italy: it corresponded to the beginning of the second phase of the lockdown. From May 18th shops reopened (as well as cafès and restaurants) and people were allowed to go outside without the previously required form justifying their reasons, and meeting up with friends was permitted. During this period students were still attending their online classes.

This cross-sectional study used structured interviews, conducted by two psychologists (GS and FS, Doctors of Clinical and Psychology). Interviews were about 10 to 20 min in duration for each student and respondents’ answers where recorded by researchers on a questionnaire form (ad hoc created) during the interviews. Students and their parents were at home during the interviews, conducted with a video-meeting platform (Zoom).

Scientific literature related to the psychological impact of quarantine was reviewed and ad-hoc interviews were created in order to investigate changes in routine of children and adolescents and psychological reactions due to the coronavirus lockdown.

### Inclusion and exclusion criteria

To be included in the study participants have to be primary or middle school students (data were collected from across all eight school year levels) living in Milan (Lombardy, Italy). Those children and their parents were recruited via different channels (newsletter, website, and social networks) of the Laboratory for Mother and Child Health of the Mario Negri Institute in Milan. Participants were excluded if they were not able or were not willing to attend the Zoom video.

### Procedure

Before conducting the interviews with study participants, a pilot study was conducted to test the interview design with four students (two males and two females of different grades): at the end participants were asked whether the questions were clear and made them comfortable. The parent was involved at the beginning of the interview in order to introduce the child and reply to a few quick questions: COVID-19 cases within the family context, developmental disorders of the child (such as specific learning disorder/special educational needs), residency, and type of school attended by the child (private/state school).

The child directly responded to the main part of the interview, which investigated:

Socio-demographic variables such as age, gender, school grade, house and family situation (information on people living with the respondent during the lockdown), and average screen time per day. Socioeconomic data, such as household income and the number of rooms in the house, were not collected.

#### Perceived changes in routine during the lockdown

- Three questions compared **distance learning** with normal schooling, and concerned: (a) difficulty in maintaining attention during online lessons than before (yes/no); (b) students’ tiredness and fatigue, for example “Compared to normal schooling, is distance schooling more tiring?” (yes/no); and (c) their motivation and commitment to remote schooling, for example “Compared to normal schooling, do you feel less committed and motivated to online school? (yes/no)
- For those who responded positively to all the above questions, adjustment to online schooling was a real struggle.

- The interviews investigated the perceived **eating habits** during quarantine: quantity (for example, “In this quarantine period you were eating as normal or more/less than you used to do before?”) and quality (“Do you eat the same things that you were used to eat before or more junk food?”) of food consumption. We focused on those subjects who were not eating the same as before and reported an increase in junk food and sweets.
- Concerning **sleeping habits**, we analysed whether there had been changes in sleep patterns or the child/adolescent was sleeping the same as before (“Do you sleep more/less than usual or the same as before?”) If there had been changes we asked if they were related to difficulties in falling asleep (“Do you have difficulties in falling asleep?“) or waking up many times during the night, or with having more nightmares than usual. Moreover, we also asked the responder if during the quarantine he or she had wished to sleep in his parents’ bed, which could be considered as a regressive behaviour.

#### Emotional and behavioural changes

Six items were adapted from the **anxiety** clinical scale of the “Trauma and symptom checklist for children” [18]. A 4-point Likert-type scale was employed to measure the anxiety level: 0 “never”, 1 “sometimes”, 2 “lots of times”, 3 “almost all the time”. We divided the scores into 3 main categories: normal anxiety (0–9), mild to moderate anxiety (10–14), and severe anxiety (15–18): a subject scored positive for anxiety if the total score was ≥ 10.

Concerning **mood symptoms**, six items were adapted from the “Short Mood and feeling questionnaire” [19] in order to investigate whether during the quarantine there had been changes. In particular, we explored if the participant felt that was crying more often than before, had lost interest in doing things that they usually enjoyed, was more irritable or tired during quarantine, reported poor concentration or had mood swings. Possible answers were 1 “yes” and 2 “no”, with a total score ranging from 0 to 6. Mood symptoms were considered clinically significant if the total score was ≥ 4 (positive scoring on at least four out of six questions).

### Statistical analyses

Data are reported as number and percentage of responders. Data analysis was performed using frequency distributions for categorical variables summarized using proportions and associations tested using chi-square or Fisher’s exact test where applicable. Continuous variables were summarized using means, standard deviations, and median.

To identify factors influencing psychological distress (anxiety and mood symptoms) we computed odd ratios (OR) considering the significance of the confidence intervals (CI).

Statistical significance was evaluated using a 95 % confidence interval and a two-tailed p-value of < 0.05. SAS software, version 9.4 (SAS, Institute Inc., Cary, NC, USA) was used for all statistical analyses.

### Ethical aspects

This study followed the criteria for reporting observational studies (STROBE, additional file 1). The ethics committee of the Besta Neurological Institute in Milan approved the study protocol (Report number 73). According to the Italian laws and good ethical practice, a written and informed consent of the parents was required via email before participation in the study. The invitation included an information leaflet explaining the nature of the interviews, who would be present and what to expect, making it clear that participants could stop the interview at any time and the link to connect at the scheduled date, and time of the video-interview. Moreover, before the interview we introduced the aims of the study to the interviewed youth and asked them consent to be involved in the present study.

## Results

### Socio-demographic characteristics of the sample

In total 82 children and adolescents, aged 6 to 14 (mean age of 10.4 years) were interviewed; of whom 53.7 % were males and 46.3 % were females.

Just over half of students (54.9 %) went to primary school and 45.1 % to middle school. Most (72 %) were attending public schools.

All students were living in Milan (Italy). In particular, 53 (64.6 %) were living in the urban area of Milan, while 29 (35.4 %) in the metropolitan area. A minority of the students (11 %) had specific learning disorders or special educational needs. Most (89 %) had brothers or sisters; the majority of them (78 %) had parents who were working during the lockdown. 68.3 % of the subjects spent less than 2 h per day on leisure screen time, but 26 respondents (31.7 %) reported spending more than two hours a day in front of screens, excluding hours of online lessons and homework.

Of the 82 children and adolescents who participated in the study, 3 (3.6 %) tested positive for SARS-CoV-2 virus and 12(14.6 %) had close relatives who had been infected. This means that 7 respondents had been exposed to COVID-19 without contracting the disease.

### Perceived changes in routine:

Concerning changes in routine during the lockdown we considered different areas:

- **Distance learning**: 65 respondents (79.3 %) reported that it was more difficult to focus during online lessons, 53 (64.6 %) found it more tiring, and almost half of the children and adolescents (47.3 %) felt less committed to remote schooling. 27 subjects (32.9 %) responded positively to all the questions related to online schooling, meaning that those students struggled to adjust to home learning.
- **Eating habits**: the perception of changes in eating habits was observed in 63.4 % of the population, mainly in primary school students (OR = 0.38, 95 % C.I: 0.152–0.966, p = .04). In particular, more than half of the sample (57.3 %) reported eating more during the lockdown, with an increase in consumption of junk food, snacks, and sweets. 36 responders (43.9 %) completely changed their dietary habits during the lockdown: they were not eating the same amount of food and were consuming more junk food.
- **Sleeping habits**: Sleep habits were also affected by the lockdown measures. 50 respondents (61 %) reported changes in sleep pattern: the majority of them had difficulties in falling asleep and woke up many times during the night. They went to bed later than before, but due to school lessons had to wake up at around the same time in the morning. Moreover, nearly half of the sample (48.8 %) reported that during the quarantine they wished to sleep in their parents’ bed. In total 23 children and adolescents (28 %) had difficulties sleeping and wished to sleep their parents’ bed.

### Fears and symptoms:

The main fear, shared by the 75.6 % of the sample, is the thought that their family members could fall ill with COVID-19. Children and adolescents were more frightened than before for their family than for themselves; only 18.3 % of the respondents replied that they were mostly terrorized by the idea of being hospitalized. 18 (22 %) subjects had normal scores of anxiety (Table 1**)**, the mean score was 11.56 (2.65 s.d.). 54 scored mild to moderate, and 10 had severe anxiety symptoms: in total 78 % reported mild to severe anxiety during quarantine. It could be that the pandemic and the related stress and vulnerability to virus could have exacerbated previous symptoms; it is even possible that those symptoms were not present before, but arose due to the isolation condition. There were no significant differences in anxiety levels between males and females, nor between primary and middle school students. None of the following characteristics (Table 1) is significantly associated with anxiety levels.

In particular, 30.5 % of the responders reported that almost all of the time they felt afraid that something bad might happen and felt afraid of the dark.

**Table 1:** Characteristics of total responders by anxiety

	Anxiety				OR	CI 95%	***p***-value
	No	%	Yes	%			
**Gender**							
Male	11	61.1	33	51.6	*Reference*		
Female	7	38.9	31	48.4	*1.48*	*0.51-4.29*	*0.4744*
**COVID_family members**							
Yes	2	11.1	10	15.6	*1.481*	*0.29-7.46*	*0.6341*
No	16	88.9	54	84.4	*Reference*		
**Class**							
Primary	11	61.1	34	53.1	*Reference*		
Middle	7	38.9	30	46.9	*1.39*	*0.48-4.03*	*0.5484*
**Type of school**							
Private	6	33.3	17	26.6	*0.723*	*0.23-2.23*	*0.573*
Public	12	66.7	47	73.4	*Reference*		
**Residential area**							
Milan, urban area	10	55.6	43	67.2	*Reference*		
Milan, metropolitan area	8	44.4	21	32.8	*0.61*	*0.21-1.77*	*0.3641*
**Brothers or sisters**							
Yes	16	88.9	57	89.1	*1.018*	*0.19-5.39*	*0.9833*
No	2	11.1	7	10.9	*Reference*		
**Special learning needs**							
Yes	3	16.7	6	9.4	*0.517*	*0.12-2.31*	*0.3883*
No	15	83.3	58	90.6	*Reference*		
**Parents working during quarantine**							
Yes	15	83.3	49	76.6	*Reference*		
No	3	16.7	15	23.4	*1.53*	*0.39-6.01*	*0.5422*
**Screen usage**							
Less than two hours	3	16.7	23	35.9	*Reference*		
More than two hours	15	83.3	41	64.1	*0.36*	*0.09-1.36*	*0.1316*
**Remote school, major difficulties**							
Yes	3	16.7	24	37.5	*3*	*0.79-1.14*	*0.1078*
No	15	83.3	40	62.5	*Reference*		
**Sleeping as normal**							
Yes	7	38.9	25	39.1	*Reference*		
No	11	61.1	39	60.9	*0.99*	*0.34-2.90*	*0.9894*
**Wish to sleep in parent’s bed**							
Yes	6	33.3	34	53.1	*2.267*	*0.76-6.78*	*0.1434*
No	12	66.7	30	46.9	*Reference*		
**Children who were sleeping differently and wished to sleep in parent’s bed**							
Yes	4	22.2	19	29.7	*1.478*	*0.43-5.07*	*0.5352*
No	14	77.8	45	70.3	*Reference*		
**Eating as normal**							
Yes	10	55.6	20	31.3	*Reference*		
No	8	44.4	44	68.8	*2.75*	*0.94-8.01*	*0.0637*
**Eating the same quality of food**							
Yes	10	55.6	25	39.1	*Reference*		
No, more junk food and sweets	8	44.4	39	60.9	*1.95*	*0.68-5.61*	*0.2154*
**Eating different quantity and quality of food**							
Yes	5	27.8	31	48.4	*2.442*	*0.78-7.65*	*0.1254*
No	13	72.2	33	51.6	*Reference*

- 36 responders (43.9 %) had significant mood symptoms (Table 2), the mean score was 3.43 (1.62 s.d.). The higher frequency of mood symptoms was mainly associated with screen usage (OR = 0.35, 95 % C.I: 0.13–0.91, p = .03) and with changes in dietary habits: in quantity (OR = 4.14, 95 % C.I: 1.51–11.35, p = .0057), quality (OR = 3.90, 95 % C.I: 1.50-10.12, p = .0051) and both (OR = 5.67, 95 % C.I: 2.18–14.74, p < .001).
- Interestingly, nearly three-quarters of the interviewed (73.2 %) reported getting angry more easily than they usually do. The majority of them (90.2 %) reported missing their friends a lot, and almost two third (72 %) reported missing their hobbies and extracurricular activities (sports or language courses) a lot.

**Table 2:** Characteristics of total responders by changes in mood

	Changes in mood						OR	CI 95%	p-value
	No (total score < 4)	***%***	Yes (total score ≥ 4)	***%***	Total	%	*Reference*		
**Gender**									
Male	24	*52.2*	20	*55.6*	44	*53.7*	*Reference*		
Female	22	*47.8*	16	*44.4*	38	*46.3*	*0.87*	*0.36-2.09*	*0.7606*
**COVID_family members**									
Yes	4	*8.7*	8	*22.2*	12	*14.6*	*3.00*	*0.82-10.92*	*0.0956*
No	42	*91.3*	28	*77.8*	70	*85.4*	*Reference*		
**Class**									
Primary	26	*56.5*	19	*52.8*	45	*54.9*	*Reference*		
Middle	20	*43.5*	17	*47.2*	37	*45.1*	*1.16*	*0.48-2.79*	*0.7353*
**Type of school**									
Private	12	*26.1*	11	*30.6*	23	*28.0*	*1.25*	*0.47-3.28*	*0.6551*
Public	34	*73.9*	25	*69.4*	59	*72.0*	*Reference*		
**Residential area**									
Milan, urban area	27	*58.7*	26	*72.2*	53	*64.6*	*Reference*		
Milan, metropolitan area	19	*41.3*	10	*27.8*	29	*35.4*	*0.55*	*0.21-1.39*	*0.206*
**Brothers or sisters**									
Yes	40	*87.0*	33	*91.7*	73	*89.0*	*1.65*	*0.38-7.11*	*0.5016*
No	6	*13.0*	3	*8.3*	9	*11.0*	*Reference*		
**Special learning needs**									
Yes	4	*8.7*	5	*13.9*	9	*11.0*	*1.69*	*0.42-6.83*	*0.459*
No	42	*91.3*	31	*86.1*	73	*89.0*	*Reference*		
**Parents working during quarantine**									
Yes	37	*80.4*	27	*75.0*	64	*78.0*	*Reference*		
No	9	*19.6*	9	*25.0*	18	*22.0*	*1.37*	*0.48-3.91*	*0.5559*
**Screen usage**									
Less than two hours	10	*21.7*	16	*44.4*	26	*31.7*	*Reference*		
More than two hours	36	*78.3*	20	*55.6*	56	*68.3*	*0.35*	*0.13-0.91*	*0.0309*
**Remote school, major difficulties**									
Yes	14	*30.4*	13	*36.1*	27	*32.9*	*1.29*	*0.51-3.26*	*0.5876*
No	32	*69.6*	23	*63.9*	55	*67.1*	*Reference*		
**Sleeping as normal**									
Yes	20	*43.5*	12	*33.3*	32	39.0	*Reference*		
No	26	*56.5*	24	*66.7*	50	61.0	*1.54*	*0.62-3.81*	*0.3511*
**Wish to sleep in parent’s bed**									
Yes	22	*47.8*	18	*50.0*	40	*48.8*	*1.09*	*0.46-2.61*	*0.8451*
No	24	*52.2*	18	*50.0*	42	*51.2*	*Reference*		
**Children who were sleeping differently and wished to sleep in parent’s bed**									
Yes	13	*28.3*	10	*27.8*	23	28.0	*0.98*	*0.37-2.58*	*0.9615*
No	33	*71.7*	26	*72.2*	59	72.0	*Reference*		
**Eating as normal**									
Yes	23	*50.0*	7	*19.4*	30	*36.6*	*Reference*		
No	23	*50.0*	29	*80.6*	52	*63.4*	*4.14*	*1.51-11.35*	*0.0057*
**Eating the same quality of food**									
Yes	26	*56.5*	9	*25.0*	35	*42.7*	*Reference*		
No, more junk food and sweets	20	*43.5*	27	*75.0*	47	*57.3*	*3.90*	*1.50-10.12*	*0.0051*
**Eating different quantity and quality of food**									
Yes	12	*26.1*	24	*66.7*	36	*43.9*	*5.67*	*2.18-14.74*	*0.0004*
No	34	*73.9*	12	*33.3*	46	*56.1*	*Reference*

A feedback on the study’s findings was provided to participants and interested people throughout a public national webinar.

## Discussion

lThis is the first study to directly investigate, from the viewpoint of Italian primary and middle school students, the impact of the COVID-19 quarantine on the perceived changes in their routine and psychological distress. Italy was, after China, the second most highly affected country at the time, with the pandemic spreading very fast around Milan, one of the most involved cities. In that period, the emergency level was very high and Italian population was living in an unusual situation. The preventive measures were very tight, people had to remain at home and it was forbidden for children to go outside to the park or meeting friends. These preventive measures were necessary to avoid the spreading of the virus, but they were very struggling for the youngest. Even if there is still significant controversy about the role of children in spreading the virus, school closures resolution showed that in that period children were considered the main spreaders [2], impacting their worries about infecting their parents and grandparents. Results of our research reveal that children were more concerned for their loved ones health condition than for their own.

For most children and youth, the normal routine was disrupted: with the implementation of social distancing interventions, direct human contact became highly restricted, with most activities that typically occupy youths’ lives – schooling, extracurricular activities, and socialization with peers – transitioning to electronic-based platforms.

Routine normally gives the young an increased feeling of safety in the context of uncertainty. Consistent evidence demonstrates that the structured environment of weekdays may help to protect children by regulating obesogenic behaviours, most likely through compulsory physical activity opportunities, restricting caloric intake, reducing screen time occasions, and regulating sleep schedules [20].

For this reason, most of the guidelines recommend creating new routines and structures each day to adapt to the pandemic situation.

Our results show that almost half of responders completely changed their dietary habits during the lockdown, eating different amounts of food and consuming more junk food. Sleep habits were also affected by the lockdown measures, with changes in sleep pattern, especially concerning difficulties in falling asleep and waking up many times during the night. Many children and adolescents also wished to sleep in their parents’ bed.

The findings of the present study are consistent with another Italian survey, completed by parents of 2 to 14 years old children [11]; they suggested that one in four children (26.5 %) showed the regressive symptom of the demand for physical proximity to their parents during the night, and almost one in five (18.2 %) manifested fears that they had never had before. Half of the children (53.5 %) showed increased irritability, intolerance to rules, whims and excessive demands, and one in five presented mood changes (21.2 %) and sleep problems, including difficulty in falling asleep, agitation, and frequent waking up (20 %). According to the literature, we can confirm our expectations among negative consequences on perceived changes habits due to the quarantine period.

The current study strongly supports the hypothesis positing that the COVID-19 pandemic will “exacerbate all of the risk factors for weight gain associated with summer recess” [14]. Specifically, a longitudinal study of children and adolescents with obesity affirmed that eating, activity, and sleep behaviors changed in an unfavorable direction 3 weeks into their confinement during the national lockdown [21].

Are changes in sleep temporary, or will a subset of youth experience longer-term sleep disturbances that originated during the COVID-19 pandemic? Will changes in sleep patterns (such as an increasingly late bedtime in adolescents) result in difficulties once normality starts to return (e.g., when schools reopen and early mornings are required once more) [15].

Moreover, the reduction of outdoor activities and social interaction may have been associated with an increase in children’s emotional and behavioral difficulties. Our findings showed mostly mild to moderate anxiety symptoms, but also significant anxiety in some youth, and nearly half of the responders reported frequent mood swings.

Compared to previous research conducted before the pandemic, this study found high levels of self-reported anxiety. The worldwide prevalence of any anxiety and depressive disorder among children according to Diagnostic and Statistical Manual (DSM) and International Statistical Classification of Diseases and Related Health Problems (ICD) was shown to be 6.5 and 2.6 % respectively [22]. However, the criteria for DSM and ICD were not used in the current study and children reported their symptoms focusing on their own perspective, compared to how they felt before the COVID-19 outbreak.

Our results reveal higher rates of perceived symptoms than those found in another survey conducted in China with primary school students during the Coronavirus-19 outbreak [9], although these studies cannot be compared due to different assessment tools.

This study suggested that 403 Chinese students (22.6 %) and 337 students (18.9 %) showed depressive and anxiety symptoms, respectively.

Our findings are not consistent with those of a Chinese study in which secondary school students yielded the highest prevalence and levels of depressive, anxiety and stress symptoms, and primary school students the lowest [23]: in the current study we didn’t find any significant difference in symptomatology between primary and middle school students. There is mounting evidence of increased mental health problems during the acute stages of COVID-19 [6, 9, 24]. For a subset of children and youth, however, the consequences of pandemic-related stress will not be immediately observable, and will only be detectable following a certain period of development.

It is important to highlight that the presence of depressive symptoms (even if sub-threshold) or psychological unease in a few children may be hidden by an apparently adaptive behavior. The effects of stress exposure may not manifest until a certain degree of neurobiological development has occurred or alterations in the social environment lead to change: new difficulties for children who initially appeared well-adapted may surface later in development. The mental health ramifications of COVID- 19 are likely to be longstanding, but not simply chronic [25]. The results confirm our expectations about psychological distress generated by the quarantine period. Moreover, these results highlight that the most important things for children and adolescents are social interactions, what they missed the most are friends, sports and school. There is a pressing need for giving importance to the children priorities.

### Strengths and limitations

There are several limitations to this study.

Socioeconomic details such as household income and number of rooms in the house were not collected, and these data could be helpful in planning future requirements with respect to quarantine. Moreover, this could be considered as a potential limit because of the findings are less generalizable and slightly representative of children in Milan.

We could not calculate how many people refused to participate because we’ve advertised the study on different channels and only those parents and children who wanted to be enrolled contacted us.

Our evaluation was conducted in the first phase of the pandemic, unfortunately baseline data (prior the COVID-19 outbreak) are not available. This has a significant impact, since we can’t guarantee that the perceived symptomatology arose in the quarantine and was not even present before. The results indicate the changes that the children, themselves perceived, but without an appropriate comparison or a baseline measure, we cannot be certain that these perceptions were actually reflected in reality. Another limitation is that our current study could not evaluate whether these outcomes will be long-lasting after the COVID-19 outbreak. It will be interesting to continue to follow up with these participants to improve our understanding about how long these outcomes will last.

### Implications and future research direction

The COVID-19 crisis highlights that school fulfils not only an educational mission of knowledge acquisition, but it also satisfies the socialisation needs of young people.

School provides a structured setting in which children can learn and develop social skills, such as self-confidence, friendship, empathy, participation, respect, gratitude, compassion, and responsibility [26].

Children facing unexpected and unknown events typically exhibit various stress reactions: resilience, the personal attributes that help children manage everything from little disappointments to big life traumas, should be nurtured and implemented by public health programs in children and teens living in areas hit by calamities such as epidemics. If properly supported by healthcare professionals, families, and other social connections, including school environment, children and adolescents can appropriately overcome a condition of distress and prospectively stabilize emotionally and physiologically [24]. In conclusion, we would like to highlight that children with pre-existing mental and physical disability are at utmost risk given the current situation [27, 28].

The challenges of online learning coupled with a lack of recreational activities that can be done at home can prove to be frustrating for children with physical disabilities. It becomes even more important to keep children with physical and mental disability not only physically safe, but also look after their psychological and emotional wellbeing. In particular, social distancing and its effects are extremely novel and difficult to understand for children, especially those experiencing developmental and intellectual delays. This affects their wellbeing and places them at a higher risk for clinically significant mental health issues [29]. Taken into consideration the small number of children with special needs included in the present study, we didn’t find any significant difference amongst children with special needs and the rest of the sample in all the considered variables. This study has highlighted the high levels of perceived distress experienced by children during the quarantine; furthermore, it is necessary to take into consideration that those symptoms are likely to be exacerbated for children with additional needs, frustrated due to disruptions in their daily routines and possible interruption of their regular therapy sessions.

### Conclusions

In summary, we found that 78 % of the Italian children aged 6 to 14 experienced anxiety symptoms due to the COVID-19 epidemic quarantine measures; nearly half of the sample (43.9 %) reported significant mood symptoms. In general, a large proportion of participants described having struggled to adjust to home learning and completely changed their dietary and sleeping habits.

Children are not indifferent to the dramatic impact of the COVID-19 epidemic: our data confirm their difficulties in adapting to the quarantine measures. The effects of stress exposure may not manifest later on during the children’s development, and, for this reason, it would be interesting to follow up on these participants to improve our understanding of how long these outcomes may last.

## Supplementary Information

**Additional file 1. **STROBE checklist. STROBE: The Strengthening the Reporting of Observational Studies in Epidemiology (STROBE) Statement: guidelines for reporting observational studies, 22 item checklist

## Data Availability

The datasets used and/or analyzed during the current study are available from the corresponding author on reasonable request.
